# Toxicity Thresholds Based on EDTA Extractable Nickel and Barley Root Elongation in Chinese Soils

**DOI:** 10.3390/ijerph15040669

**Published:** 2018-04-04

**Authors:** Guangyun Zhu, Bao Jiang, Guohang Yang, Jumei Li, Yibing Ma

**Affiliations:** 1 School of Resources and Environment, University of Jinan, Jinan 250022, China; zhuguangyun1993@163.com (G.Z.); yangguohang888@163.com (G.Y.); 2 Institute of Agricultural Resources and Regional Planning, Chinese Academy of Agricultural Sciences, Beijing 100089, China; jiangbao@caas.cn (B.J.); lijumei@caas.cn (J.L.)

**Keywords:** EDTA, nickel, risk assessment, soil

## Abstract

The uncertainty in the risk assessment of trace metal elements in soils when total metal contents are used can be decreased by assessing their availability and/or extractability when the soils have a high background value or different sources of trace metal elements. In this study, the added water-soluble nickel (Ni) toxicity to barley root elongation was studied in 17 representative Chinese soil samples with and without artificial rainwater leaching. The extractability of added Ni in soils was estimated by three sequential extractions with ethylenediaminetetraacetic acid (EDTA). The results showed that the effective concentration of EDTA extractable Ni (EC_50_), which caused 50% inhibition of barley root elongation, ranged from 46 to 1019 mg/kg in unleached soils and 24 to 1563 mg/kg in leached soils. Regression models for EDTA extractable Ni and total Ni added to soils against soil properties indicated that EDTA extractable Ni was significantly correlated with the total Ni added to soils and that pH was the most important control factor. Regression models for toxicity thresholds based on EDTA extractable Ni against soil properties showed that soil citrate dithionate extractable Fe was more important than soil pH in predicting Ni toxicity. These results can be used to accurately assess the risk of contaminated soils with high background values and/or different Ni sources.

## 1. Introduction

Most current soil risk assessment and soil quality criteria for trace metal elements are based on their total concentrations in soil [1]. However, the biological toxicity of trace metal elements is hard to identify if total metal concentrations are used to assess ecological risk [2]. Therefore traditional soil quality criteria for trace metal elements may not be suitable when attempting to assess the ecological risk of soils with high background values and/or different input sources of trace metal elements. These inaccuracies could give rise to underestimations or overestimations of trace metal pollution [3] because total concentrations of trace metal elements could not represent metal toxicity well [4]. Previous results suggested that it was more accurate to estimate the toxicity of trace metal elements in soils by measuring extractable fractions rather than total concentrations [5,6]. 

The concentrations, forms and extractability of trace metal elements generally determined their mobility, availability and toxicity. There are many different methods are used to assess the different chemical forms and fractions of trace metal elements in soils [7]. Generally, trace metal elements in soils are fractionated operationally by sequential extraction procedures into water-soluble plus exchangeable, specifically adsorbed on soil particle surfaces, and fractions bound to carbonates, Fe(Al)-Mn oxides, organic matter, and primary and layer silicates [8]. In the sequential extraction procedures, a chelating reagent, ethylenediaminetetraacetic acid (EDTA), was used to remove the specifically adsorbed (inner-sphere surface complexes) fraction [8,9,10]. Also, an EDTA extraction protocol for trace metal elements in soils and certified reference soil materials were developed by the Measurements and Testing Programme (formerly BCR) of the European Commission [11,12]. When comparing EDTA extractability of Zn and Mn with two sequential extraction procedures (modified Tessier [13] and proposed by the Community Bureau of Reference (BCR) [14]), it was found that in the term of phytoavailability predictions the sequential extractions are not better than EDTA single extraction [15]. For different fractions of trace metal elements in soils, the water-soluble plus exchangeable fractions are considered to be readily available; the specific adsorbed fraction, which can be extracted by powerful chelating reagents, can be potentially available; and fractions bound to carbonates, Fe(Al)-Mn oxides, organic matter and minerals are generally hard to be taken up by plants [10,16]. Therefore, the fractions of water-soluble, exchangeable and specifically adsorbed trace metal elements in soils may be important to their availability/toxicity, especially for risk assessment in contaminated soils. 

Single chemical extraction methods are most commonly used to evaluate the availability of trace metal elements [10,17,18]. The major extractants for estimating availability/toxicity presently in use are unbuffered and buffered salt solutions, dilute acidic solutions, and solutions containing chelating agents [18]. Diluted acidic solutions, such as 0.43 mol/L HNO_3_ (ISO 17586-2016) [19] has been used, which partially dissolve trace metal elements associated with different fractions such as exchangeable, carbonates, iron and manganese oxides and organic matter [20]. However, there are some limitations for diluted acidic solutions in high calcareous soils. The salt solutions, such as 0.01 mol/L CaCl_2_, 1 mol/L NH_4_NO_3_, were also used to extract exchangeable trace metal elements in soils. The exchangeable fraction includes weakly adsorbed elements retained on soil solid surfaces by relatively weak electrostatic interactions, elements that can be released by ion-exchange processes and elements that can be coprecipitated with carbonates [18]. The powerful chelating agents, such as diethylenetriamine pentaacetic acid (DTPA) and EDTA, have been used widely in soil extractions for predicting the availability/toxicity of trace metal elements in soils. Quevauviller [21,22] reported that 0.005 mol/L DTPA (pH 7.3) was less suitable for Cr and Ni than other metals and EDTA was found to be the method of preference. Echevarria et al. [23] found that Ni in the plant of red clover came from the pool of the isotopically exchangeable Ni in soil, and in their experimental conditions, DTPA extracted mainly the isotopically exchangeable Ni. However, some of the Ni chelated by EDTA was not fully isotopically exchangeable [24]. Gupta and Sinha [25] studied the heavy metal accumulation in sesame using different single extractants such as EDTA, DTPA, NH_4_NO_3_, CaCl_2_ and NaNO_3_ in soil amended with sludge. Accordingly, among this group of reagents, EDTA extraction is widely used to quantify the labile pool or to predict the available pool [26]. However, because of the competition with soil constituents (e.g., organic matter), a single-step EDTA extraction procedure may underestimate the potentially toxicity of trace metal elements in soil, multiple sequential EDTA extractions may be a suitable method in the assessment of their potential ecological risk. 

In the present study, the toxicity of added water-soluble nickel (Ni) to barley root elongation was studied in 17 representative Chinese soil samples with and without leaching by artificial rainwater. The extractability of Ni in soil samples were analyzed with three sequential EDTA extractions. The aims of the study were to develop a method for soil risk assessment based on the extractability of trace metal elements in soils instead of total metal concentrations. In particular, we sought to (1) determine the relationships between EDTA extractable Ni, total added Ni and soil properties; and (2) develop quantitative relationships between soil properties and toxicity thresholds based on EDTA extractable Ni in soils. 

## 2. Materials and Methods 

### 2.1. Soil Samples and Treaments

Seventeen soils (0–20 cm depth) from multiple locations in China were chosen as representatives of major Chinese soil types. The soil pH and organic matter content distributions were consistent with agricultural soils in China. The soil property ranges (Table 1) were as follows: pH 4.93 to 8.90; organic carbon content (OC) 0.60% to 4.28%; cation exchange capacity (CEC) 6.36 cmol^+^/kg to 33.59 cmol^+^/kg, and clay content 10% to 66%. 

Air-dried soil samples (<2 mm) were spiked with NiCl_2_ solution and each soil sample set consisted of eight added Ni concentration treatments. Soils with pHs < 5, from 5 to 7, and >7 were spiked at the rates of 12.8–800, 25–1600, and 37.5–2400 mg/kg Ni, respectively. After equilibrating at 100% maximum water-holding capacity for 2 d, the spiked soils were left to air-dry and then were sieved again through a 2-mm plastic mesh. 

Half of the spiked soil samples were leached by artificial rainwater [27] to overcome potential salinity effects and to simulate natural precipitation. This treatment reduced the difference in Ni speciation between laboratory-treated and field-aged soils. The artificial rainwater used for leaching consisted of 5 × 10^−4^ M calcium chloride, 5 × 10^−4^ M calcium nitrate, 5 × 10^−4^ M magnesium chloride, 10^−4^ M sodium sulfate, and 10^−4^ M potassium chloride at pH 5.9. Details about the leaching treatment can be found in Li et al. [28]. The leached soil samples were also air-dried and sieved through a 2-mm plastic mesh before use. The total Ni concentrations in the unleached and leached soils were taken from Li et al. [28], and were obtained using atomic absorption spectroscopy (ZEEnit 700, Analytik Jena, Jena, Germany) according to Zarcinas et al. [29]. 

### 2.2. Extraction of Added Ni in Soils 

Soil extraction was carried out using EDTA, which is one of the chemical reagents widely used in fractionation and single extraction procedures, 0.05 mol/L EDTA extraction is widely used to quantify the labile pool or to predict the available pool [7,25,26] The soil samples (5 g each, air-dried, <2 mm) were combined with 25 mL of 0.05 mol/L EDTA in 50 mL polypropylene centrifuge tubes and shaken for 2 h at room temperature (~20 °C) on a reciprocating shaker. The soil suspension was centrifuged at about 4000 r/min for 20 min, after which the supernatants were passed through a syringe filter with a pore size of 0.45 μm. Another 25 mL EDTA was then added to the residue to continue the extraction. The extraction step was repeated three times over 6 h which resulted in stable Ni levels in the samples. The EDTA extractable Ni concentrations in each extraction were measured by using atomic absorption spectroscopy (ZEEnit 700; Analytik Jena, Jena, Germany). There were two replicates for each treatment. 

### 2.3. Barley Root Elongation Assay 

The barley root elongation assay was performed according to ISO 11269-1 [30]. Detailed procedures for the barley root elongation assay and barley root elongation data acquisition can be found in Li et al. [28]. The barley root elongation percentage with respect to the controls (RE, %) in a test medium was calculated using the equation:

(1) $$\mathrm{RE}=\frac{{\mathrm{RE}}_{t}}{{\mathrm{RE}}_{c}}\times 100\%$$

where RE_t_ is root length in the test medium and RE_c_ is root length in the control, which had not been spiked with Ni. 

### 2.4. Statistical Analysis

The dose-response data were fitted by a log-logistic curve [31]

(2) $$Y=\frac{Y_{0}}{1+e^{b\left(X-M\right)}}$$

where Y is relative barley root elongation (%), X is the measured EDTA extractable Ni (mg/kg) after log10 transformation, M is EC_x_ (effective concentration of EDTA extractable Ni that decreases barley root elongation at EC_10_, EC_20_, or EC_50_ after log10 transformation, and Y_0_ and b are curve fitting parameters. 

The EC_10_, EC_20_, and EC_50_ values were based on the measured EDTA extractable Ni concentrations in soils and were estimated from the respective log-logistic dose-response curves. The 95% confidence intervals (95% CI) for the EC_10_, EC_20_, and EC_50_ values were calculated according to Haanstra et al. [31], and were obtained from the fitted curve parameters data. 

A stimulation response that occurs at low doses, but is inhibited at higher doses is defined as hormesis, and was modeled according to Schabenberger et al. [39] using Tablecurve 2D V5.01 (Systst Software Inc, San Jose, CA, USA). The EC_10_, EC_20_, and EC_50_ values, with their respective 95% CI, were determined as follows:

(3) $$Y=\frac{a+\mathrm{bX}}{1+\left[\frac{k}{100-k}+\left(\frac{100}{100-k}\right)\frac{\mathrm{bc}}{a}\right]e^{\mathrm{dln}\left(X/c\right)}}$$

where Y is barley root elongation; X is the measured EDTA extractable Ni concentration; a, b, c, and d, are curve fitting parameters; and k is a variable that is related to the effective concentrations (e.g., EC_10_ and EC_50_). Further details about the hormesis analysis can be found in Guo et al. [40] and Li et al. [28]. When k equals 10, 20, or 50, the parameter c is defined as the EC_10_, EC_20_, or EC_50_ value, respectively, and its confidence interval can be calculated by Tablecurve 2D V5.01. The significance level for hormesis is detected by parameter b and the hormesis response is considered to be significant when the 95% confidence intervals of parameter b are above zero. 

Stepwise multiple linear regressions were calculated using SPSS 19.0 for Windows (SPSS, Chicago, IL, USA) and were used to examine the relationship between toxicity thresholds (EC_10_, EC_20_, and EC_50_). They were based on the measured EDTA extractable Ni concentrations and soil property parameters, which were only log transformed if the normality and homogeneity of variance tests suggested they should be. Relationships were deemed significant at *p* ≤ 0.05.

## 3. Results and Discussion 

### 3.1. Toxicity Thresholds for Nickel in Soils Based on EDTA Extractions 

The EC_10_, EC_20_ and EC_50_ values for the unleached soils varied from 3 (Haikou soil) to 426 mg/kg (Hangzhou soil), from 6 (Haikou soil) to 510 mg/kg (Hangzhou soil); and from 22 (Haikou soil) to 722 mg/kg (Lingshan soil), respectively, which represents 142-, 85-, and 33-fold variations among the soils when one single EDTA extraction was used. The EC_10_, EC_20_ and EC_50_ values for the leached soils varied from 2 to 789 mg/kg, from 4 to 839 mg/kg, and from 13 to 1013 mg/kg, respectively, which represents 395-, 210-, and 78-fold variations, respectively, among the soils when one single EDTA extraction was used. The wide variations in EC_10_, EC_20_ and EC_50_ values indicated that the EDTA extraction could not accurately predict Ni toxicity in soils. Therefore, the soil properties have to be taken into account when the EDTA extraction method is used to predict Ni toxicity in soils. 

Hormesis was observed in non-leached Chongqing soil and leached Guangzhou soil. One of the EC_10_ values, three of the EC_20_ values, and six of the EC_50_ values in leached calcareous soils (pH ≥ 8.2) could not be calculated because the leaching reduced or eliminated the toxicity caused by the added Ni in the soils (Table 2). The concentrations of one single EDTA extractable Ni in these calcareous soils ranged from 2 to 732 mg/kg. However, there was no Ni toxicity in the soils after leaching. Similar results were also found by Li et al. [28] when the EC_x_ values were expressed as the total Ni added to these calcareous soils. 

The toxicity thresholds at the three inhibition levels for barley root elongation estimated by three sequential EDTA extractions in unleached and leached soils are listed in Table 3. The EC_10_, EC_20_, and EC_50_ values increased by 83%, 103%, and 46%, respectively, in leached soils compared with the results with one single EDTA extraction, and by 136%, 151%, and 185% in unleached soils, respectively. Similarly wide variations in EC_x_ values were also found in other studies when three sequential EDTA extractions were used. The orders for the EC_x_ values in this study were EC_10_ (103-fold) > EC_20_ (60-fold) > EC_50_ (22-fold) for unleached soils and EC_10_ (335-fold) > EC_20_ (142-fold) > EC_50_ (65-fold) for leached soils. 

### 3.2. The Influence of Leaching on Ni Toxicity 

In leached soils, there was a significant (*p* ≤ 0.05) decrease in toxicity of a single EDTA extractable Ni in six soils (35%) at the EC_10_ level, 10 soils (59%) at the EC_20_ level, and 13 soils (76%) at the EC_50_ level; when three sequential EDTA extractions were used, there was also a decrease in toxicity of the three EDTA extractable Ni in five soils (29%) at the EC_10_ level, eight soils (47%) at the EC_20_ level, and 10 soils (59%) at the EC_50_ level (Table 2 and Table 3). The leaching effects on decrease in Ni toxicity depended on the soil properties. Leaching had a comparatively stronger influence on alkaline soils than on acidic and neutral soils (Table 2 and Table 3).

The EC_x_ values based on the single and three EDTA extractions, the leaching treatment decreased the toxicity of soluble metal salts in soils. Oorts et al. [27] showed the Ni toxicity effects on soil microbial processes were decreased by leaching to a greater extent in an alkaline soil (pH 7.6) than that in acidic and neutral soils (pH 4.5–6.1) when the EC_x_ values are expressed as total added Ni in soils. Furthermore, Li et al. [28] also found that the greatest decrease in Ni toxicity to barley root elongation induced by leaching occurred in soils where the pH ≥ 8.2, which was consistent with the results produced in this study. It has been suggested that leaching alleviates or eliminates Ni toxicity in soils by reducing the pH and salinity [28]. Jiang et al. [41] reported that when the pH increased by 1, Ni phytotoxicity decreased 2.60-fold (1.86–3.72) and that this was due to the formation of Ni precipitates on solid surfaces. Furthermore, the maximum added Ni concentration (3200 mg/kg) in field soils from Dezhou, Shandong did not lead to a significant decrease in wheat growth. However, leaching decreased Ni toxicity when the EC values were expressed as EDTA extractable Ni in soils. That is to say, EDTA extractable Ni represents potential toxicity. It is not an indicator of toxicity. When EDTA extractable Ni is selected as a risk assessment criterion, the effects of soil properties cannot be ignored.

### 3.3. The Relationships between EDTA Extractable and Total Ni as Affected by Soil Properties 

The extractability of Ni added to soils by EDTA was expressed as the proportion (%) extracted relative to the total Ni added to the soils. One single EDTA extraction can extract about 46% of the total Ni added to the leached and unleached soils; and three EDTA extractions can extract about 70–74% on average. 

**Figure 1:**
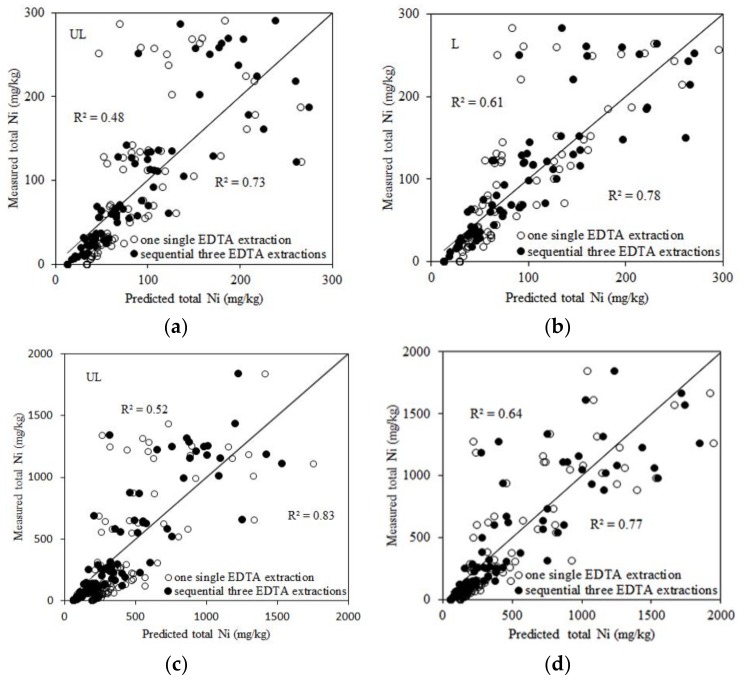
The relationships between the measured and predicted total Ni. (**a**) calculated from Equations (1) and (3) in Table 4; (**b**) from Equations (5) and (7) in Table 4; (**c**) from Equations (9) and (11) in Table 4; and (**d**) from Equations (13) and (15) in Table 4. UL and L represent unleached and leached soils, respectively.

Linear regressions were used to study the relationships between EDTA extractable Ni and total Ni added to soils as affected by soil properties. The total Ni added to soils segregated into two ranges: 0–300 mg/kg and 0–2000 mg/kg (Table 4). The relationships between EDTA extractable Ni and soil properties, such as pH, OC, and CEC, could be good predictors of total Ni added to soils. When the total Ni concentration added to soils was under 300 mg/kg, Ni and pH and one EDTA extraction explained 48% to 65% of the variance in total Ni added to the unleached and leached soils. Also, Extractable Ni with three EDTA extractions and pH could predict the total Ni in soils with an R^2^ = 0.73 for unleached soils and an R^2^ = 0.78 for leached soils. When the total Ni concentration added to soils was 2000 mg/kg, extractable Ni with three EDTA extractions and pH could predict total Ni in soils with an R^2^ = 0.83 for unleached soils and an R^2^ = 0.77 for leached soils. Incorporating OC or CEC into the regression models for unleached and leached soils led to a small or no improvement in predictability for total Ni added to soils when one and three EDTA extractions were used. The predictive ability of the linear regression equations based on three EDTA extractions is better than that based on one single EDTA extraction for unleached and leached soils. The measured total Ni and the predicted total Ni are shown in Figure 1. The high correlations found between total Ni added to soils, EDTA extractable Ni, and soil properties indicates that EDTA extractable Ni can be used as an alternative to the total Ni added to soils, which means that the disadvantages associated with using total Ni as a risk assessment criterion can be overcome. 

The toxicity of metals in soils depends on many factors, including their speciation, pH, organic matter content, cation exchange capacity, and soil texture [42]. In this study, soil pH was the most important factor affecting the relationships between EDTA extractable Ni and the amounts that were actually added to the soils. Cui et al. [43] also reported that pH was negatively correlated with the concentrations of leachable, available, and bioaccessible copper (Cu) and cadmium (Cd). This was probably because soil pH is one of the most important factors controlling the sorption and mobility of trace metal elements in soils [44,45]. The results from this study showed that three EDTA extractions improved the accuracy and stability of the prediction models for EDTA extractable Ni in soils. Furthermore, EDTA extractable Ni in soils better expresses the trace metal elements toxicity than total Ni.

### 3.4. Toxicity Thresholds Based on EDTA Extractable Ni in Soils as a Function of Soil Properties 

Simple and multiple stepwise regressions were carried out between Ni toxicity thresholds (EC_10_, EC_20_, and EC_50_ values-based EDTA extractable Ni) and soil properties (Table 5 and Table 6). Although there is generally less statistical uncertainty for EC_50_ than EC_10_ and EC_20_ [46], the effects of soil properties on EC_10_ and EC_20_ values are listed in Table 5 and Table 6 because they are important when deciding risk assessment and ecological criteria for Ni in soils. 

The results with one EDTA extraction in unleached soils showed that when CEC or OC were used as the single factor in the regression models, CEC was found to explain 33% of the variance in the EC_10_ values, and OC could explain 32% to 36% of the variance in the EC_20_ and EC_50_ values. However, when two factors were used, such as CEC and citrate dithionate extractable (CD)Mn or OC and CDAl in the regression models, the predictability of the regression models improved significantly, with R^2^ = 0.54 for EC_10_ and R^2^ = 0.66 for EC_50_. Furthermore, when OC, CDAl, and OXFe (oxalate extractable Fe) were incorporated into the regression models, the multiple linear regressions were further improved, with R^2^ = 0.72 for EC_20_. For leached soils, CDFe was found to be the best single factor for predicting Ni toxicity at the EC_10_ (R^2^ = 0.42), EC_20_ (R^2^ = 0.64), and EC_50_ (R^2^ = 0.73) levels. Fe, Al, and Mn oxides, CaCO_3_, and pH factors could also improve the models. Furthermore, when four factors (Fe, Al and Mn oxides, and CaCO_3_) were incorporated, the R^2^ for the regression model was 0.99 for the EC_50_ values. 

When the soil toxicity thresholds were based on extractable Ni with three EDTA extractions, CDFe was the most important single factor in predicting Ni toxicity in the unleached and leached soils. For unleached soils, CDFe as the single factor could explain 39% of the variance in the EC_10_ values, 47% of the variance in the EC_20_ values, and 62% of the variance in the EC_50_ values. Incorporating OC into the regression models for unleached soils gave a significant, but slightly smaller, improvement in predictability for the EC_50_ and EC_20_ thresholds. When three factors (CDFe, OC, and OXFe) were introduced into the regression models, the predictability of the regression models improved, with R^2^ = 0.82 for EC_10_ and R^2^ = 0.86 for EC_20_. For leached soils, CDFe as the single factor could explain 52% of the variance in the EC_10_ values, 74% of the variance in the EC_20_ values and 75% of the variance in the EC_50_ values. Factors such as OXFe, CDMn, and OXAl, improved the predictability of the regression models. When four factors (CDFe, OXFe, CDMn, and OXAl) were incorporated, the regression models had an R^2^ value of = 0.98 for the EC_50_ thresholds.

When the different regression equations were compared (Table 5 and Table 6), it was found that pH occasionally affected the toxicity thresholds expressed as extractable Ni based on one EDTA extraction, but that this effect disappeared when three EDTA extractions were used; this suggests that the soil constituents are more important than soil pH. However, whether one or three extractions were used, the EDTA extractable Ni as an indicator still needs to consider the effects of soil properties probably because most of EDTA extractable Ni is potentially available, not instantly available, to plants. The adjusted coefficients of determination for the EC_50_ predictive equations were higher than for the EC_10_ and EC_20_ predictive equations, which was probably due to the higher statistical uncertainty of the EC_10_ and EC_20_ values. Furthermore, the adjusted coefficients of determination for the unleached soil EC_50_ predictive equations were lower than those for the leached soils, which could be due to the high salinity and low pH induced by the addition of water soluble Ni salts to the soil.

### 3.5. Applications in Risk Assessment and Ecological Criteria Setting 

Current soil quality criteria for trace metals are based on total metal concentrations, regardless of their availability and/or extractability in soils with high trace metal background values or their trace metal sources. Therefore, they do not assess the potential toxicity of trace metals and could lead to either underestimations or overestimations of trace metal pollution. 

In this study, the relationships between total Ni added to soils and EDTA extractable Ni as affected by soil properties were set up. Furthermore, the regression models, based on EDTA extractions as a function of soil properties, accurately predicted Ni toxicity. These results provide a basis for establishing soil environmental quality criteria based on EDTA extractable Ni. Further study on the establishment of soil environmental quality criteria based on EDTA extractable Ni will improve the accuracy of Ni ecological risk assessments.

## 4. Conclusions

In this study, EDTA extractable Ni was significantly correlated with the total Ni added to soils, and soil pH was the most important factor affecting the relationships between EDTA extractable Ni and the actual Ni added to the soils. Three sequential EDTA extractions improved the prediction accuracy and stability of toxicity models based on EDTA extractions. The regression models for toxicity thresholds based on EDTA extractable Ni against soil properties showed that soil citrate dithionate extractable Fe was more important than soil pH when attempting to predict Ni toxicity in soils. These results showed that EDTA extractable Ni was better at assessing potential Ni toxicity in soils than the total Ni in a soil. Determining the extractability of soil trace metal elements is a key process in ecological risk assessment. Therefore, the creation of an extraction procedure that can determine the toxicity of soil trace metal elements will improve the accuracy of ecological risk assessments. 

## Figures and Tables

**Table 1:** Main properties of the soils used in this study.

Silt Name	pH ^a^	CEC ^b^	OC ^c^	CaCO_3_	CD ^d^ Al	CDFe	CDMn	OX ^e^ Al	OXFe	OXMn
	(1:5)	(cmol^+^/kg)	(%)	(%)	(mg/kg)	(mg/kg)	(mg/kg)	(mg/kg)	(mg/kg)	(mg/kg)
Haikou, Hainan	4.93	8.75	1.5	<0.5	9478	83,920	574	1736	1337	200
Qiyang, Hunan	5.31	7.47	0.9	<0.5	3293	26,154	422	1326	1146	294
Hailun, Heilongjiang	6.56	33.6	3	<0.5	1244	6559	396	1954	3298	451
Jiaxing, Zhejiang	6.7	19.3	1.4	<0.5	794	10,824	297	1106	6212	261
Hangzhou, Zhejiang	6.8	12.83	2.5	<0.5	631	8413	153	1003	4980	135
Chongqing, Sichuan	7.12	22.3	1	<0.5	370	7727	315	603	989	283
Guangzhou, Guangdong	7.27	8.3	1.5	0.15	1488	11,411	48	532	1811	33
Lingshan, Beijing	7.48	22.6	4.3	4.27	706	6950	276	1304	1697	267
Hulunber, Neimeng	7.66	22.7	2.7	0.27	956	5259	322	1441	2477	307
Gongzhuling, Jilin	7.82	28.7	2.2	0.27	1067	6932	366	1786	1447	387
Shijiazhuang, Hebei	8.19	11.7	1	3.84	579	7544	261	734	826	222
Urumchi, Xinjiang	8.72	10.3	0.9	5.08	396	4795	305	551	600	251
Yangling, Shanxi	8.83	8.46	0.6	8.92	461	7193	350	863	707	288
Langfang, Hebei	8.84	6.36	0.6	2.42	273	3729	112	291	537	74
Zhengzhou, Henan	8.86	8.5	1.6	0.15	361	4289	166	482	581	121
Zhangye, Gansu	8.86	8.08	1	7.75	451	8356	331	674	1980	233
Dezhou, Shandong	8.90	8.33	0.7	6.17	369	4965	219	497	644	145

^a^ Measured in deionized water (soil: solution ratio 1:5) [32]. ^b^ Cation exchange capacity was determined using the ammonium chloride method [32]. ^c^ Organic carbon: the difference between the total and inorganic carbon contents [33,34]. ^d^ Citrate dithionate extractable metal [35,36]. ^e^ Oxalate extractable metal [37,38].

**Table 2:** Toxicity thresholds based on the concentrations of a single EDTA extractable Ni and barley root elongation in unleached and leached soils.

Site	Unleached Soil (mg/kg)			Leached Soil (mg/kg)		
	EC_10_ (95% CI)	EC_20_ (95% CI)	EC_50_ (95% CI)	EC_10_ (95% CI)	EC_20_ (95% CI)	EC_50_ (95% CI)
Haikou, Hainan	3 (1–7)	6 (3–13)	22 (14–35)	2 (1–4)	4 (2–7)	13 (10–19)
Qiyang, Hunan	11 (4–30)	21 (10–44)	63 (44–91)	6 (3–12)	14 (8–26)	65 (46–93)
Hailun, Heilongjiang	384 (170–869)	431 (250–743)	527 (477–582)	384 (247–596)	504 (365–695)	803 * (620–1039)
Jiaxing, Zhejiang	136 (88–210)	147 (115–187)	167 (151–185)	414 * (319–537)	522 * (435–627)	776 * (715–843)
Hangzhou, Zhejiang	426 (370–491)	510 (462–563)	692 (656–731)	734 * (643–837)	827 * (751–909)	1013 * (956–1075)
Chongqing^a^, Sichuan	71 (57–88)	87 (73–105)	124 (104–149)	48 (35–66)	77 (61–97)	171 (148–197)
Guangzhou^a^, Guangdong	92 (78–107)	103 (89–117)	124 (108–142)	789 (97–6443)	839 * (162–4349)	933 * (391–2224)
Lingshan, Beijing	324 (244–430)	435 (354–536)	722 (646–806)	331 (283–387)	436 (387–491)	698 (641–761)
Hulunber, Neimeng	341 (177–655)	424 (272–661)	617 (466–817)	594 (491–718)	715 (618–828)	982 * (893–1080)
Gongzhuling, Jilin	175 (6–4968)	179 (18–1789)	186 (111–312)	353 (298–419)	389 (349–435)	459 * (434–468)
Shijiazhuang, Hebei	106 (90–125)	141 (127–158)	231 (215–248)	NC * (-)	NC * (-)	NC * (-)
Urumchi, Xinjiang	32 (11–90)	47 (21–106)	93 (52–169)	27 (1–752)	≥ 263 * (-)	NC * (-)
Yangling, Shanxi	7 (2–21)	13 (6–28)	34 (20–58)	31 (0–12630)	≥ 295 * (-)	NC * (-)
Langfang, Hebei	98 (61–158)	130 (96–178)	212 (164–274)	659 * (272–1594)	676 * (350–1305)	706 * (538–927)
Zhengzhou, Henan	21 (14–33)	39 (29–52)	109 (92–130)	≥ 650 * (-)	NC * (-)	NC * (-)
Zhangye, Gansu	212 (195–232)	243 (229–258)	305 (297–314)	≥ 480 *(-)	NC * (-)	NC * (-)
Dezhou, Shandong	20 (4–111)	37 (11–129)	106 (47–240)	67 (17–268)	≥ 180 * (-)	NC * (-)

EC_x_ (x = 10, 20, or 50): The effective extractable Ni concentration when one single EDTA extraction was used that decreased barley root elongation by 10%, 20%, or 50% compared with the control. 95% CI: ± 95% confidence interval. -: The 95% CI could not be determined. NC: Toxicity thresholds could not be calculated because the Ni dose resulted in over 50% inhibition. ^a^ Significant hormesis effect on barley root elongation in unleached and leached soils. * Significant difference between unleached and leached EC_10_, EC_20_, or EC_50_ values after using a t-test at the *p* ≤ 0.05 significance level.

**Table 3:** Toxicity thresholds based on the Ni concentrations of three EDTA extractions and barley root elongation in unleached and leached soils.

Site	Unleached Soil (mg/kg)			Leached Soil (mg/kg)		
	EC_10_ (95% CI)	EC_20_ (95% CI)	EC_50_ (95% CI)	EC_10_ (95% CI)	EC_20_ (95% CI)	EC_50_ (95% CI)
Haikou, Hainan	6 (2–16)	12 (6–27)	46 (30–71)	4 (2–8)	8 (4–13)	24 (18–32)
Qiyang, Hunan	17 (7–43)	31 (16–60)	85 (61–119)	12 (6–24)	25 (15–44)	92 (66–129)
Hailun, Heilongjiang	617 (20–19226)	645 (65–6426)	697 (491–988)	538 (367–789)	702 (531–927)	1104 (894–1363)
Jiaxing, Zhejiang	202 (126–326)	242 (189–310)	329 (266–408)	457 * (345–604)	586 * (482–713)	897 * (823–978)
Hangzhou, Zhejiang	603 (530–685)	718 (656–786)	970 (924–1019)	1011 * (897–1138)	1125 * (1032–1226)	1352 * (1282–1425)
Chongqing^a^, Sichuan	130 (113–151)	154 (136–174)	203 (181–228)	83 (60–116)	125 (97–161)	252 (212–300)
Guangzhou^a^, Guangdong	179 (138–231)	214 (171–268)	292 (233–366)	1091 (100–11906)	1137 (175–7400)	1220 * (454–3283)
Lingshan, Beijing	428 (327–559)	569 (468–692)	927 (834–1029)	521 (451–602)	668 (598–747)	1024 (945–1110)
Hulunber, Neimeng	582 (304–1115)	715 (459–1115)	1019 (782–1327)	943 (778–1144)	1137 (980–1319)	1563 * (1419–1721)
Gongzhuling, Jilin	549 (-)	551 (-)	555 (-)	483 (407–574)	578 (514–649)	784 (733–840)
Shijiazhuang, Hebei	183 (153–219)	301 (265–341)	702 (652–756)	NC * (-)	NC * (-)	NC * (-)
Urumchi, Xinjiang	140 (39–509)	236 (90–617)	574 (323–1020)	72 (1–3551)	≥ 1058 * (-)	NC * (-)
Yangling, Shanxi	27 (8–84)	53 (23–119)	169 (98–290)	84 (0–178003)	≥ 1232 * (-)	NC * (-)
Langfang, Hebei	237 (132–425)	322 (216–480)	544 (418–707)	915 (252–3320)	941 (360–2457)	987 (662–1470)
Zhengzhou, Henan	129 (65–255)	269 (174–417)	949 (739–1219)	≥ 1339 *(-)	NC * (-)	NC * (-)
Zhangye, Gansu	310 (259–370)	389 (344–439)	574 (543–606)	≥ 81 * (-)	NC * (-)	NC * (-)
Dezhou, Shandong	40 (5–328)	86 (18–404)	321 (117–879)	217 (58–807)	≥ 648 * (-)	NC * (-)

EC_x_ (x = 10, 20, and 50): The effective extractable Ni concentration when three EDTA extractions were used that decreased barley root elongation by 10%, 20%, or 50% compared to the control. 95% CI: ± 95% confidence interval. -: The 95% CI could not be determined. NC: Toxicity thresholds could not be calculated because the Ni dose resulted in over 50% inhibition. ^a^ Significant hormesis of barley root elongation in unleached and leached soil. * Significant difference between unleached and leached EC_10_, EC_20_, or EC_50_ values after using a t-test at the *p* ≤ 0.05 significance level.

**Table 4:** Linear regressions between EDTA extractable Ni and total Ni added to soils as affected by soil properties.

Extraction Times	Equation	Unleached Soils	R^2^	Equation	Leached Soils	R^2^
	0 mg/kg < Y < 300 mg/kg					
1	(1)	Y = 34.327 + 0.168 X_1_ pH	0.48	(5)	Y = 29.535 + 0.180 X_1_ pH	0.65
	(2)	Y = 53.515 + 0.177 X_1_ pH + 0.832 CEC − 21.473 OC	0.52	(6)	Y = 52.940 + 0.186 X_1_ pH − 0.6023 CEC − 10.421 OC	0.65
3	(3)	Y = 13.270 + 0.140 X_1_ pH	0.73	(7)	Y = 13.737 + 0.144 X_1_ pH	0.78
	(4)	Y = 27.806 + 0.146 X_1_ pH + 0.832 CEC − 17.774 OC	0.75	(8)	Y = 34.725 + 0.148 X_1_ pH − 0.525 CEC − 9.324 OC	0.78
	**0 mg/kg < Y < 2000 mg/kg**					
1	(9)	Y = 194.31 + 0.270 X_1_ pH	0.52	(13)	Y = 128.160 + 0.239 X_1_ pH	0.64
	(10)	Y = 109.957 + 0.296 X_1_ pH + 4.719 CEC − 195.611 OC	0.57	(14)	Y = 217.158 + 0.244 X_1_ pH + 4.483 CEC − 100.658 OC	0.65
3	(11)	Y = 64.690 + 0.200 X_1_ pH	0.83	(15)	Y = 61.226 + 0.186 X_1_ pH	0.77
	(12)	Y = 165.479 + 0.204 X_1_ pH + 7.747 CEC − 139.907 OC	0.85	(16)	Y = 156.497 + 0.190 X_1_ pH + 3.797 CEC − 98.418 OC	0.78

Y = Total Ni added to soils; X_1_ = EDTA extractable Ni; R^2^ = coefficient of determination.

**Table 5:** Simple and multiple linear regressions between Ni toxicity thresholds based on the Ni concentrations (mg/kg) of one EDTA extraction and selected soil properties.

No.	Regression Equations	R_adj_^2^	*p*				
	**Unleached Soils (*n* = 17)**						
1	Log EC10 = − 0.062 + 1.722 log CEC	0.326	0.010				
2	Log EC10 = 2.595 + 2.226 log CEC − 1.335 log CDMn	0.543	0.001	0.013			
3	Log EC20 = 1.795 + 1.360 log OC	0.319	0.011				
4	Log EC20 = 4.161 + 1.751 log OC − 0.837 log CDAl	0.611	<0.001	0.004			
5	Log EC20 = 2.043 + 1.156 log OC − 0.927 log CDAl + 0.783 log OXFe	0.723	0.011	0.001	0.023		
6	Log EC50 = 2.074 + 1.090 log OC	0.360	0.006				
7	Log EC50 = 3.896 + 1.392 log OC − 0.644 log CDAl	0.660	<0.001	0.002			
	**Leached Soils**						
8	Log EC10 = 8.678 − 1.659 log CDFe (*n* = 16)	0.419	0.004				
9	Log EC10 = 5.638 − 1.961 log CDFe + 1.339 log OXFe (*n* = 16)	0.698	<0.001	0.003			
10	Log EC10 = 7.420 - 1.945 log CDFe + 1.516 log OXFe − 1.044 log CDMn (*n* = 16)	0.836	<0.001	<0.001	0.005		
11	Log EC20 = 8.813 − 1.636 log CDFe (*n* = 14)	0.640	<0.001				
12	Log EC20 = 6.525 − 1.783 log CDFe + 0.905 log OXFe (*n* = 14)	0.827	<0.001	0.003			
13	Log EC20 = 7.651 − 1.572 log CDFe + 0.869 log OXFe − 0.765 log CDMn (*n* = 14)	0.915	<0.001	<0.001	0.006		
14	Log EC20 = 1.720 - 0.881 log CDFe + 1.220 log OXFe - 0.666 log CDMn + 0.252 pH (*n* = 14)	0.944	0.019	<0.001	0.005	0.033	
15	Log EC20 = 1.658 − 0.957 log CDFe + 0.906 log OXFe − 1.100 log CDMn + 0.258 pH + 0.795 log OXAl (*n* = 14)	0.967	0.004	0.001	0.001	0.010	0.028
16	loge C50 = 8.207 − 1.408 log CDFe (*n* = 11)	0.727	0.001				
17	log EC50 = 5.707 − 1.392 log CDFe + 0.745 log OXFe (*n* = 11)	0.879	<0.001	0.008			
18	Log EC50 = 6.214 − 1.209 log CDFe + 0.787 log OXFe − 0.573 log CDMn (*n* = 11)	0.966	<0.001	<0.001	0.002		
19	Log EC50 = 6.787 − 1.593 log CDFe + 0.755 log OXFe − 0.614 log CDMn + 0.385 log CDAl (*n* = 11)	0.982	<0.001	<0.001	<0.001	0.032	
20	Log EC50 = 6.342 − 1.555 log CDFe + 0.819 log OXFe − 0.616 log CDMn + 0.406 log CDAl + 0.043 CaCO3 (*n* = 11)	0.993	<0.001	<0.001	<0.001	0.005	0.023

R_adj_^2^ = adjusted coefficient of determination; *p* = significance level of the factors included in the regression equations.

**Table 6:** Simple and multiple linear regressions between Ni toxicity thresholds based on extractable Ni concentrations (mg/kg) of three EDTA extractions and selected soil properties.

No.	Regression Equations	R_adj_^2^	*p*			
	**Unleached soils (*n* = 17)**					
1	Log EC10 = 6.864 − 1.198 log CDFe	0.389	0.004			
2	Log EC10 = 4.341 − 1.460 log CDFe + 1.130 log OXFe	0.766	<0.001	<0.001		
3	Log EC10 = 5.191 − 1.396 log CDFe + 0.748 log OXFe + 0.746 log OC	0.823	<0.001	0.012	0.037	
4	Log EC20 = 6.676 − 1.109 log CDFe	0.472	0.001			
5	Log EC20 = 6.670 − 1.146 log CDFe + 1.115 log OC	0.795	<0.001	<0.001		
6	Log EC20 = 5.519 − 1.251 log CDFe + 0.700 log OC + 0.516 log OXFe	0.856	<0.001	0.014	0.021	
7	Log EC50 = 6.355 − 0.956 log CDFe	0.616	<0.001			
8	Log EC50 = 6.350 − 0.980 log CDFe + 0.719 log OC	0.844	<0.001	<0.001		
	**Leached soils**					
9	Log EC10 = 8.945 − 1.666 log CDFe (*n* = 16)	0.521	0.001			
10	Log EC10 = 6.566 − 1.901 log CDFe + 1.048 log OXFe (*n* = 16)	0.721	<0.001	0.005		
11	Log EC10 = 8.178 − 1.887 log CDFe + 1.208 log OXFe − 0.945 log OXMn (*n* = 16)	0.859	<0.001	<0.001	0.003	
12	Log EC20 = 9.365 − 1.709 log CDFe (*n* = 14)	0.741	<0.001			
13	Log EC50 =8.187 − 1.362 log CDFe (*n* = 11)	0.751	<0.001			
14	Log EC50 = 5.952 − 1.348 log CDFe + 0.666 log OXFe (*n* = 11)	0.882	<0.001	0.010		
15	Log EC50 = 6.387 - 1.191 log CDFe + 0.701 log OXFe − 0.492 log CDMn (*n* = 11)	0.950	<0.001	0.001	0.011	
16	Log EC50 = 6.257 − 1.251 log CDFe + 0.449 log OXFe − 0.897 log CDMn + 0.717 log OXAl (*n* = 11)	0.984	<0.001	0.004	<0.001	0.007

R_adj_^2^ = adjusted coefficient of determination; *p* = significance level of the factors included in the regression equations.
